# Foreign Correspondence

**Published:** 1863-08

**Authors:** 


### FOREIGN CORRESPONDENCE.

LONDON, June 30th, 1863.

Prof. E. INGALS, M. D.,—*My Dear Doctor*, I embrace this my earliest leisure moment to fulfill my promise to write you from London. By a hasty look at medical matters here one can but be fully impressed with the fact that the material for clinical study is abundant, for in a city which contains three millions of people, and has so many well organized Hospitals in one or other of which every variety of disease is treated, every facility is offered to the medical student.

In some respects the London Hospital is favorably situated especially for the student of Surgery. It is in a part of the city where accidents are of frequent occurrence, such as fractures, dislocations, contusions, &c., and I am told that it is not an infrequent circumstance for students to leave other hospitals for a time and go to the London to familiarize themselves with the mode of treating these injuries, which they are in the habit of styling "the study of coarse surgery." Mr. Luke, who was for so many years connected with this hospital, and was among its most active officers, has retired, and devotes himself to a lucrative private Surgical practice. Mr. Critchet has also just resigned the office of Surgeon here, and has been succeeded by the appointment of Mr. Hutchinson, whose ad vancement is spoken of as remarkable, he having been first elected assistant Surgeon only about four years since.

One of the oldest and largest hospitals in the city is St. Bartholomew's. Here Mr. Paget is one of the leading Surgeons. He is a neat and dextrous operator. I noticed on his operating day that he prefers the circular to the flap operation in amputations of the thigh. As a lecturer he is agreeable, clear, concise, and graphic. A considerable portion of this hospital is appropriated to the diseases peculiar to females, and is under the direction of Dr. Greenhalgh, who was elected

Professor of Obstetrics, &c., about a year and a half ago. Having so recently entered upon the discharge of his duties in so large an institution, the Doctor is, as we might expect, industrious, vigilant and enthusiastic in the profession, and I may justly add he is a successful physician. The opportunity afforded him for testing the various methods of treating the numerous diseases to which females are subject, is quite unlimited, and is embraced to the fullest extent. The incision of the neck of the uterus for the cure of dysmenorrhœa is just at present the practice that seems to attract considerable attention at St. Bartholomew's, the results of the practice are represented as being highly satisfactory. While there is nothing new in the principles of this practice, its general adoption would hardly meet the approbation of the judicious physician in private practice. The Actual Cautery is frequently resorted to here, in cases of ulcerated os uteri, and with benefit in some cases, but I was led to infer that much annoyance was sometimes experienced by the extension of inflammation to the peritoneum, or causing pelvic cellulitis. The line between Medical and Surgical practice is distinctly marked here, as an instance a patient with procidentia uteri had obtained no relief from any of the numerous appliances resorted to, it was then decided to extend the perineum by causing a portion of the vulva to unite, which the obstetrician was willing to undertake, but etiquette required that the patient be removed to the surgical ward.

At the University College Hospital I witnessed the operation of Lithotomy by Mr. Errichsen, which was performed rapidly, but it is not usual that such troublesome hemorrhage is met with, as in this case. In the Obstetric department Dr. Murphy still gives the lectures as he has done for twenty years. His style is plain and his teaching practical. He has just issued the second edition of his book, which is considerably more comprehensive than the first, embracing now the main topics of a general course. Here the practice differs from that followed in some other places in London; the hysterotome and the actual cautery are less frequently brought into requisition, much attention is given to correcting the *general health*, with the effect of removing local symptoms, and the same is true of the practice in this department in St. George's Hospital, where Dr. Lee denounces in no measured terms, the "cutting and burning" as French innovations which ought not to be countenanced.

The Samaritan Hospital is devoted to the treatment of diseases of females. Here a number of interesting cases may always be seen. The Surgeon of this institution, T. Spencer Wells, Esq., is just now doing considerable in the way of Ovariotomy. I witnessed the extirpation of a large ovarian tumor by him yesterday and was struck with the embarrassments which are liable to arise in this operation, even with a dexterous operator. I was unable to learn the per-centage of success of the operation here, but I find that the "Surgeons" have not adopted it as one of the ordinary operations. I met with eminent men here who represent the mortality from Ovariotomy as "fearful."

In the "London Surgical Home," which receives none but female patients, I saw several interesting cases. This is the institution where I. Baker Brown performs most of his operations. He attempted the restoration of the recto-vaginal septum on the day of my visit, which was the second attempt upon the same patient. He promised that one more operation would make the case complete. His operations are spoken of by the profession as being generally successful, without any novelty in the mode of their performance.

I was greatly pleased with my visit to the "National Hospital for Paralysis and Epilepsy," which is attended by Dr. Brown Sequard, who is without question the "right man in the right place." At this institution may be seen a large number of patients affected with various chronic diseases of the nervous centres. I was forcibly impressed with the careful manner of conducting the examinations of patients, the critical analysis of symptoms, and the candor and frankness in announcing opinions when difficulty arose. Every physician of any considerable experience, is fully aware of the obscurity of many cases of this class of diseases. I have seldom met a man in the profession whose acquaintance produced a more favorable impression upon my mind, than Dr. Brown Seguard. I found him an agreeable gentleman, as he is a profound medical scholar and successful physician.

At my visits to the "Museum of the Royal College of Surgeons" I could but notice the remarkable beauty, as well as the great number of preparations, many of them the work of the celebrated John Hunter, illustrating Anatomy, Physiology, Pathology, &c., &c., to study which would require many weeks of industrious application. As I could not study all, you will not be surprised that I devoted most of my time to the Obstetric department, which is so extensive and complete that little is left to be desired.

The medical lectures from certain chairs are given here during the summer season, a number of which I had the pleasure of hearing. While I was prepared to find less interest on the part of the classes of students, than I had been accustomed to see in the United States, I was surprised to hear a very good lecture, well *written*, read to a class of but eight students in the great city of London. When the number present was somewhat larger, I found that the roll was called, and each student was obliged to answer to his name under penalty.

I can not avoid the reflection that it is unfortunate for the student when the Professor is led by party prejudice or personal inclination to give but a partial view of a subject; that the discussion of a subject under such circumstances, however spirited, or however much talent and learning may be displayed, is unprofitable to the tyro, is not problematical.

My visit to London has been one of uninterrupted pleasure as well as profit, and the remembrance of it will ever excite in my mind the liveliest emotions of obligation for the uniform kindness and courtesy extended to me by the medical gentlemen with whom I met.

I leave to-morrow for Paris.

Truly yours, M.

